# Activation of chloramphenicol biosynthesis in *Streptomyces venezuelae* ATCC 10712 by ethanol shock: insights from the promoter fusion studies

**DOI:** 10.1186/s12934-016-0484-9

**Published:** 2016-05-20

**Authors:** Olga N. Sekurova, Jianhai Zhang, Kåre A. Kristiansen, Sergey B. Zotchev

**Affiliations:** Department of Pharmacognosy, University of Vienna, 1090 Vienna, Austria; Department of Biotechnology, Norwegian University of Science and Technology, 7491 Trondheim, Norway

**Keywords:** *Streptomyces*, Antibiotic biosynthesis, Environmental stress, Regulation, Promoter fusions

## Abstract

**Background:**

*Streptomyces venezuelae* ATCC 10712 produces antibiotics chloramphenicol (Cml) and jadomycin (Jad) in response to nutrient limitation and ethanol shock (ES), respectively. Biosynthesis of Cml and Jad was shown to be reciprocally regulated via the action of regulatory proteins JadR1 and JadR2 encoded by the *jad* cluster, and mechanism of such regulation has been characterized. However, detailed analysis of the regulatory mechanism controlling Cml biosynthesis is still lacking.

**Results:**

In the present study, several promoters from the *cml* cluster were fused to the reporter gene *gusA*. Reporter protein activity and Cml production were assayed in the wild-type strain with and without ES, followed by similar experiments with the *jadR1* deletion mutant. The latter gene was earlier reported to negatively control Cml biosynthesis, while serving as a positive regulator for the *jad* cluster. A double deletion mutant deficient in both *jadR1* and the *cml* cluster was also constructed and used in promoter fusion studies. Analyses of the results revealed that ES activates Cml biosynthesis in both wild-type and *jadR1* deletion mutant, while Cml production by the latter was ca 80 % lower.

**Conclusions:**

These results contradict earlier reports regarding the function of JadR1, but correlate well with the reporter activity data for some promoters, while reaction of others to the ES is genotype-dependent. Remarkably, the absence of Cml production in the double mutant has a profound effect on the way certain *cml* promoters react to ES. The latter suggests direct involvement of Cml in this complex regulatory mechanism.

**Electronic supplementary material:**

The online version of this article (doi:10.1186/s12934-016-0484-9) contains supplementary material, which is available to authorized users.

## Background

Biosynthesis of secondary metabolites in Gram-positive bacteria of the genus *Streptomyces* is controlled at several levels, involving global, pleiotropic, and pathway-specific regulators. Altogether, these regulators represent an intricate network that switches on biosynthesis of secondary metabolites, which is energy- and resource-demanding process, in response to particular environmental stimuli. The latter can be nutrient deprivation, addition of toxic chemicals, phage infection, co-cultivation with various organisms, temperature shift etc [1]. In *Streptomyces venezuelae* ATCC 10712, biosynthesis of antibiotic chloramphenicol (Cml) was reported to be initiated in a medium limited in nitrogen sources [2], while another type of antibiotic, jadomycin (Jad), was only produced in a culture subjected to phage infection, temperature shift or ethanol shock [3].

**Fig. 1:**
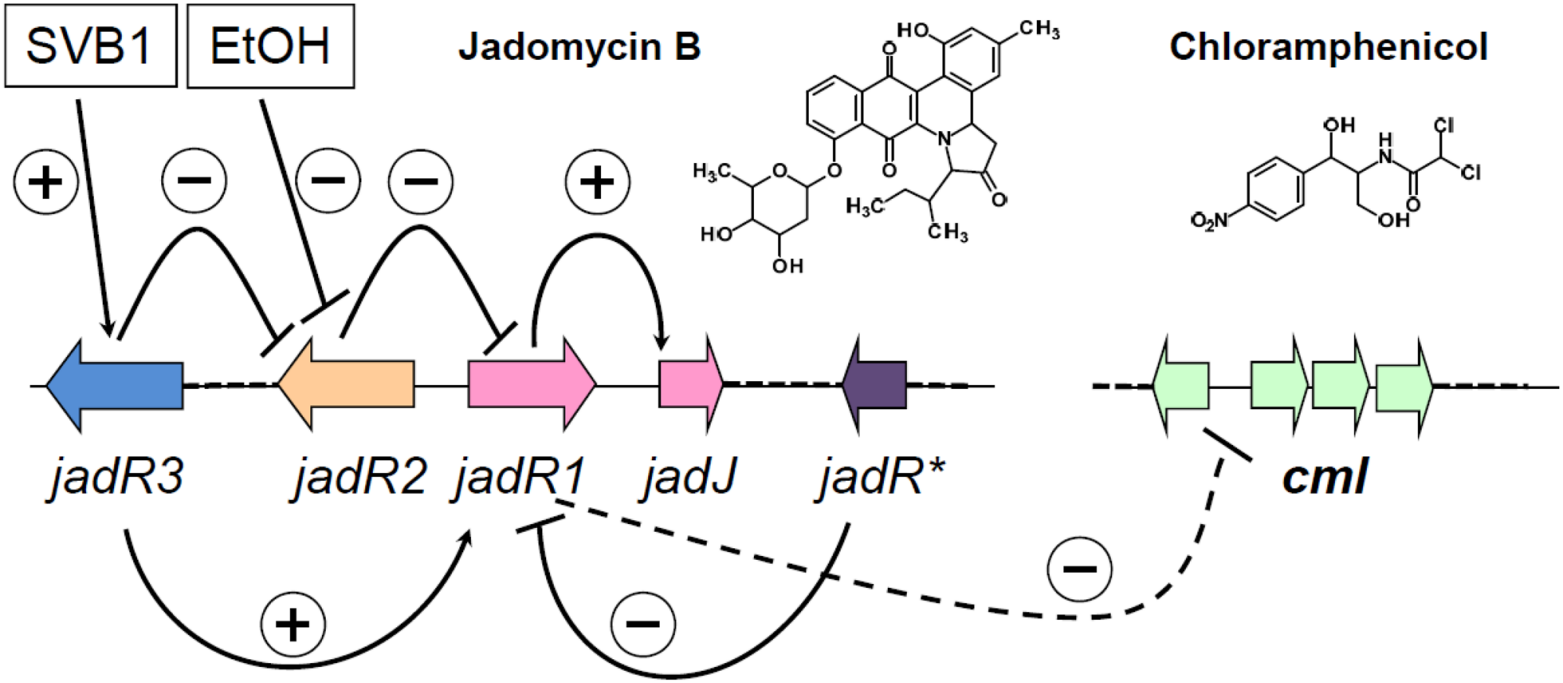
Schematic presentation of the current view on the regulation of jadomycin and chloramphenicol biosynthesis based on the reports from the Tan group [8–12]. See text for details

The intriguing mechanism behind regulation of Jad biosynthesis has recently been revealed, and involves a complex interaction between four regulatory proteins, JadR1, JadR2, JadR3 and JadR* encoded within the *jad* biosynthetic gene cluster [4–8]. According to the proposed model (Fig. 1), γ-butyrolactone SVB1 modulates DNA binding activity of JadR3, a true γ-butyrolactone receptor (GBLR), which then stimulates transcription of *jadR1* while repressing *jadR2*. The latter gene encodes a protein which, in cooperation with JadR*, represses *jadR1* transcription. It was further shown that JadR1 acts as a positive regulator for the *jad* cluster, while at the same time suppressing Cml biosynthesis. In the galactose-isoleucine (GI) medium supporting Cml production, ethanol shock (ES) somehow alleviates repression of *jadR1* gene by JadR2 and JadR*, thus allowing *jad* cluster to be expressed, while expression of the *cml* gene cluster is repressed [5]. Tan group has also demonstrated that JadR2 represents a “pseudo” GBLR that binds Jad and, less efficiently, Cml as ligands. Such binding, especially in the case of Jad, inhibits association of JadR2 with target DNA regions, thereby stimulating *jadR1* expression and generating a feedback autoregulation loop [5].

For a long time, it was believed that *cml* gene cluster does not have its own pathway-specific regulator. However, Bibb group [9] has recently reported identification of *cmlR*, a gene encoding pathway-specific regulator for the *cml* gene cluster located at its left border (Fig. 3). In this report, Cml production could not be detected in *S. venezuelae*, and *cmlR* transcription level was very low in the conditions tested, suggesting the latter being the main reason for Cml non-producing phenotype. Heterologous expression of the *cml* cluster in *Streptomyces coelicolor* was demonstrated, and deletion of *cmlR* led to complete abrogation of Cml biosynthesis, thus confirming the role of CmlR as a positive regulator and implying that in *S. venezuelae cmlR* is repressed.

In the current study, we demonstrate that Cml production in *S. venezuelae* is ES-inducible in particular growth conditions, and show response of different *cml* promoters to ES using reporter gene *gusA*. The aim of this study was to investigate whether certain promoters in *S*. venezuelae can be used for development of a new bacterial “chassis” for inducible gene expression. Interestingly, responses of certain promoters to ES were shown to be dependent on the medium, the presence of *jadR1*, and the ability of the strain to produce Cml. Taking together, these new data suggest the existence of a previously underappreciated complex network regulating Cml biosynthesis in response to environmental stimuli.

## Results

### Ethanol shock activates chloramphenicol biosynthesis in *Streptomyces venezuelae*

Production of Cml by *S. venezuelae* ATCC 10712 (ISP5230) was reported to be stimulated by deprivation of easily assimilated nitrogen sources, such as ammonia [10], and could not be detected at all in rich media containing such nitrogen sources as yeast extract [11]. Moreover, it was shown that in the GI medium supporting Cml biosynthesis, addition of 6 % v/v ethanol within 6–11 h after culture inoculation (ethanol shock, ES) suppresses Cml production while simultaneously triggering Jad biosynthesis. According to the currently accepted model for reciprocal regulation of Cml and Jad biosynthesis, no Cml production is possible upon ES due to the repression of *cml* promoters by the JadR1 regulatory protein [4, 5]. We reasoned that this phenomenon can be media-dependent, and carried out ES experiments in rich MYM medium normally used to prepare inoculum for *S. venezuelae*. The differences in the GI and MYM media are quite profound, especially with respect to carbon (galactose in GI and maltose in MYM) and nitrogen sources (isoleucine in GI and yeast extract in MYM). Starting culture of the wild-type strain was prepared by inoculating spores in TSB medium and incubating cultures for 16 h. Next, 20 mL of MYM medium buffered with MOPS was inoculated with 2 mL starting culture, followed by 8 h of growth at 30 °C (220 rpm), addition of 1.2 mL of water (negative control) or absolute ethanol. The cultures were incubated in the same conditions for 48 h after ES, and Cml in culture supernatants were assayed using UHPLC-MS/MS (see “Methods” section).

**Fig. 2:**
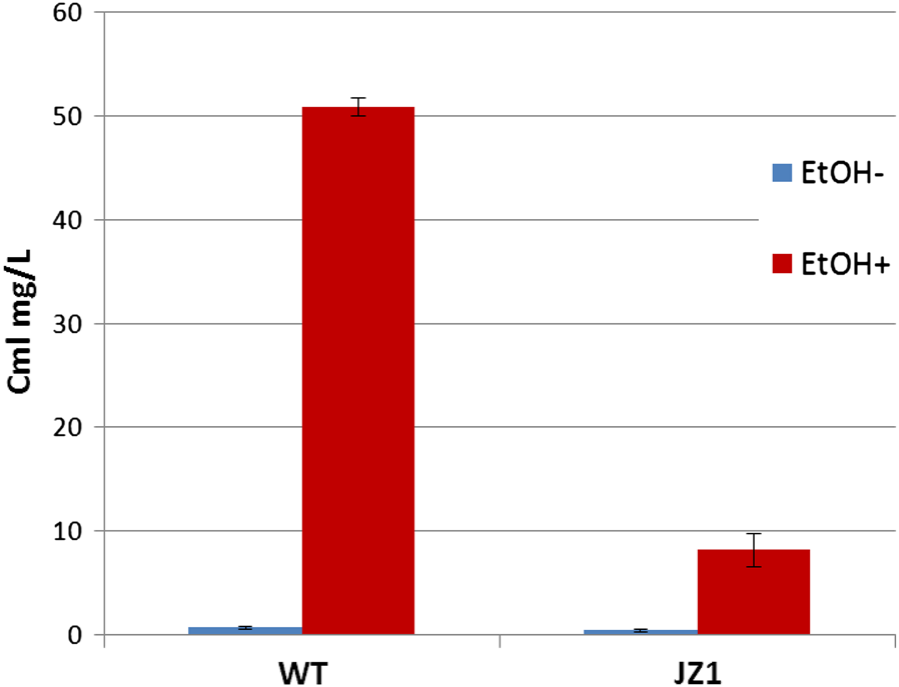
Effect of the ethanol shock on chloramphenicol production in *S. venezuelae* wild-type strain and *jadR1* deletion mutant JZ1 grown in MYM medium. Data presented are from 3 biological replicates, showing standard deviation

Surprisingly, and contradictory to the results reported for the GI medium, ES significantly stimulated Cml production, from ca 0.6 to 50 mg/L (Fig. 2). The experiment was repeated two more times, generating the same result. Next, we decided to assess the role of JadR1 regulator postulated to act as a repressor of Cml biosynthesis in the observed phenomenon. An in-frame deletion of the *jadR1* gene was constructed in the wild-type strain, generating mutant JZ1. The latter, together with the wild-type strain was used in the ES experiment in MYM medium as described above. Although Cml production was also induced by ES in the JZ1 mutant, the level of production was ca 6 times lower compared to the wild-type strain (Fig. 2). The latter contradicted the postulated repressor function of JadR1 for Cml biosynthetic pathway, suggesting that its role as a positive or negative regulator may vary depending on growth conditions. Indeed, earlier reports on the repressor role of JadR1 utilized GI medium that normally supports Cml biosynthesis [4, 5].

### *cmlR*p promoter is silent in MYM medium and does not respond to ES

As the next step toward understanding the phenomenon of Cml biosynthesis stimulation by ES, a series of promoter probe vectors were constructed. pSOK808 plasmid was assembled based on the integrative vector pSOK804 [12] with *gusA* reporter gene encoding β-glucuronidase [13] under control of a strong constitutive promoter ermE*p (see “Methods” section for details). The vector is based on the phage VWB integration system, and integrates into a chromosomal *attB* site, thus representing a stably maintained, single-copy genetic element. pSOK808 was introduced into the wild-type *S. venezuleae*, and GusA activity measured with and without ES using same growth conditions as for the Cml production in MYM (see above). Very strong GusA activity was detected in the cell extracts both with and without ES, confirming utility of the reporter plasmid.

**Fig. 3:**
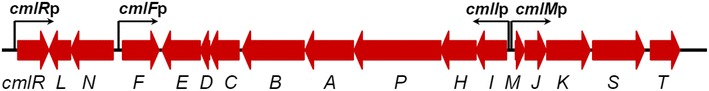
Organization of chloramphenicol biosynthesis gene cluster according to Fernandez-Martinez et al. [13]. *cml* promoters used to study the expression of *cmlF*, *cmlI* and *cmlM* genes in this study are indicated

Next, several intergenic regions apparently containing promoters were amplified from the *cml* gene cluster (Fig. 3) and used to replace the ermE*p promoter in pSOK808. In particular, DNA fragments encompassing promoters for *cmlR* (transcriptional regulator), *cmlF* (MFS transporter), *cmlI* (N-oxygenase), and *cmlM* (acyl carrier protein) genes were used. The promoter replacement was done using Gibson assembly [14] allowing seamless fusion of the promoter-Shine-Dalgarno regions with the *gusA* reporter gene.

Based on the data from Fernanded–Martinez et al. [9], who demonstrated activation of Cml biosynthesis upon overexpression of *cmlR* in *S. venezuelae*, we expected that ES positively affects expression from the *cmlR*p promoter. To test this hypothesis, p808cmlRp construct was introduced into *S. venezuelae* wild-type strain and JZ1 mutant, and the resulting recombinant strains were studied for response to ES in the same conditions as for the assessment of Cml biosynthesis. The only difference in experimental setup was that samples for measuring the GusA activity were taken 2, 8, 17 and 24 h after ES. Surprisingly, we could not detect any GusA activity in recombinant strains carrying *gusA* under control of the *cmlR*p promoter, whether they were subjected to ES or not (data not shown). Fernanded–Martinez et al. [9] carried out RT-PCR for several *cml* genes, including *cmlR,* in the wild-type *S. venezuelae*, showing low-level expression of *cmlR* in the glucose-yeast extract-malt extract medium. The expression level was somewhat higher for most of the *cml* genes upon expression of *cmlR*, but the difference was not dramatic, probably reflecting much lower Cml production level (ca 3 mg/L) compared to what we observed upon ES in the MYM medium (ca 50 mg/L). Taking all the above into account, it was logical to assume that in *S. venezuelae* grown in the MYM medium *cmlR* does not play a crucial role in regulating Cml biosynthesis, since we could not detect any *cmlR*p promoter activity even after ES.

### Response of *cml* and *jad* promoters to ethanol shock is medium- and genotype-dependent

In order to understand better the reason why Cml is produced by the MYM-grown ethanol-shocked cultures in the absence of any detectable *cmlR*p activity, *S. venezuelae* recombinant strains based on the wild type strain and JZ1 mutant carrying pSOK808 derivatives with *gusA* under control of *cmlF*p, *cmlI*p, and *cmlM*p promoters were constructed. These promoters were chosen for the following reasons: (i) CmlF is a transporter presumably responsible for the efflux of Cml from the cell; (ii) according to the bioinformatics analysis, *cmlI*p promoter appears to be responsible for the expression of *cmlI*, *cmlH*, *cmlP* and *cmlA* genes that encode enzymes critical for assembly of the Cml scaffold [9, 15]; (iii) c*mlM*p promoter is presumably driving expression of at least CmlM, an acyl carrier protein, which is indispensable of the Cml biosynthesis. The recombinant strains harbouring reporter constructs were studied in the same experimental conditions as described for the pSOK808::*cmlR*p-carrying clones (see above), and GusA activity was assayed. It shall be noted that we could not detect any increase in GusA activity after 17 h in all strains tested. Considering the fact that GusA is extremely stable protein, this most likely reflects cessation of transcription. Consequently, only GusA activities at 17 h time point were taken into consideration when assessing promoter activities. Summary of the results of these experiments is presented in Table 1.

**Table 1:** Effects of ES on the activity of *S. venezuelae* promoters depending on the medium and genetic background

Promoter	Wild-type		JZ1		JZ2	
	MYM	GI	MYM	GI	MYM	GI
*cmlF*p	1.7 up	NT	10 down	NT	2.5 up	NT
*cmlI*p	2 up	Not active	No change	No activity	No change	13 up
*cmlM*p	1.6 up	3.8 down	3 up	1.3 up	No change	1.7 up
*cmlR*p	Not active	NT	Not active	NT	Not active	NT
*jadJ*p	NT	14 up	NT	No activity	NT	No activity

Fold change in the GusA activity after ES, either up or down, are given

*NT* not tested

**Fig. 4:**
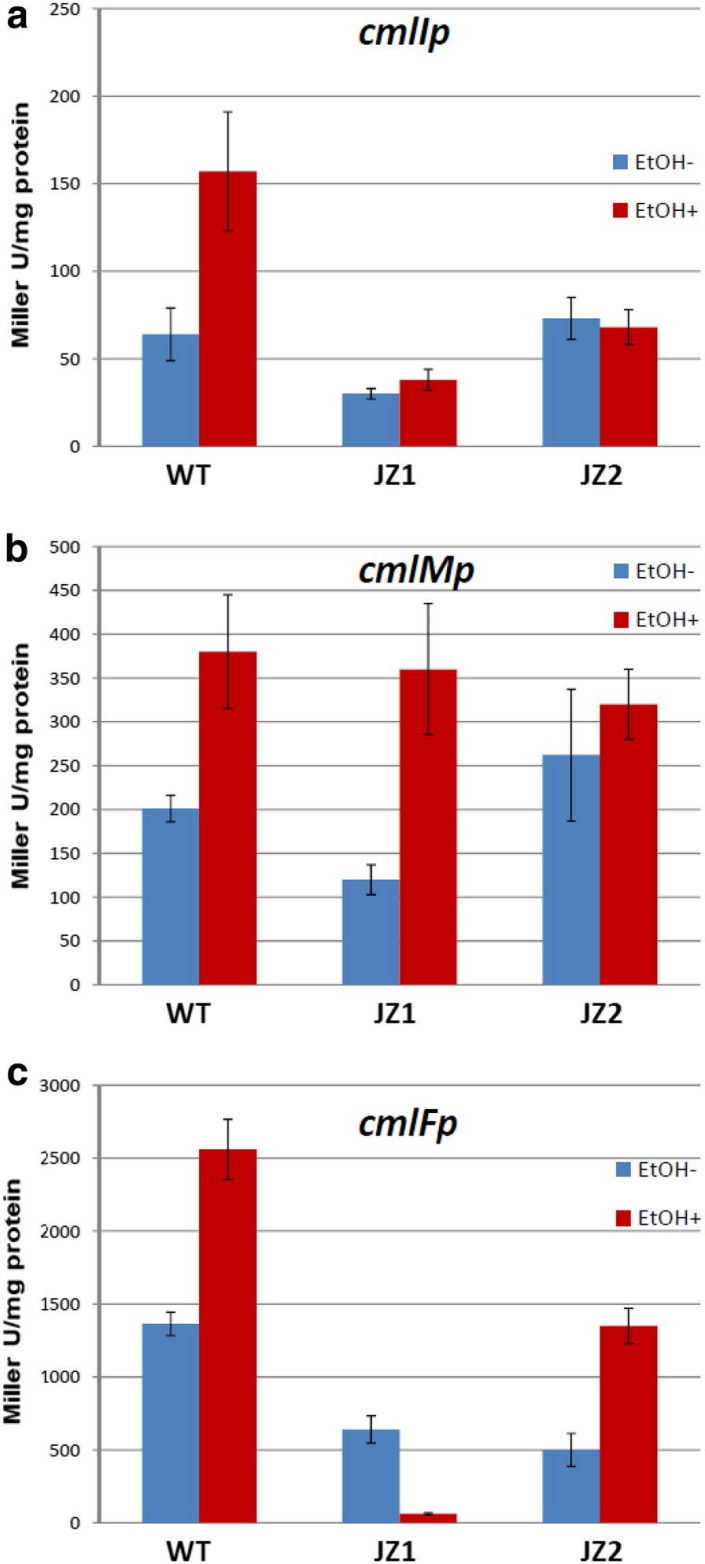
Effect of the ethanol shock on the activity of *cmlI*p (**a**), *cmlM*p (**b**) and *cmlF*p (**c**) promoters in the wild-type strain and JZ1 (Δ*jadR1*) and JZ2 (Δ*jadR1* Δ*cml*) mutants grown in MYM medium. Data presented are from 3 biological replicates, showing standard deviation

In the wild type strain, all the *cml* promoters studied were stimulated by ES to approximately same extent (ca. 40–60 %). The strongest stimulation was shown for the expression of the *gusA* reporter from the *cml* promoters in the *jadR1* deletion mutant JZ1, as well as their response to ES, were drastically different from those in the wild-type strain (Fig. 4). The levels of *gusA* expression from *cmlF*p, *cmlI*p and *cmlM*p promoters in JZ1 in the absence of ES were considerably lower compared to the wild-type strain, which correlated well with lower production of Cml by this mutant (Fig. 2). *cmlF*p activity was suppressed almost tenfold by ES, *cmlI*p activity not affected, while *cmlM*p activity stimulated ca threefold upon ES (Fig. 4). Combined, these data strongly suggest that JadR1 plays an important role as a positive regulator of Cml biosynthesis if *S. venezuelae* is grown in the MYM medium, but its absence can be partially counteracted by ES via an unknown mechanism.

We were also interested in whether ES in MYM medium stimulates expression from the *jadJ*p promoter crucial for expression of the *jad* cluster, as was shown by Xu et al. in GI medium [5]. In contrast to the latter study, we could not detect any stimulation of *jadJ*p promoter by ES in MYM medium, and the background expression of the reporter gene from this promoter remained very low (data not shown).

**Fig. 5:**
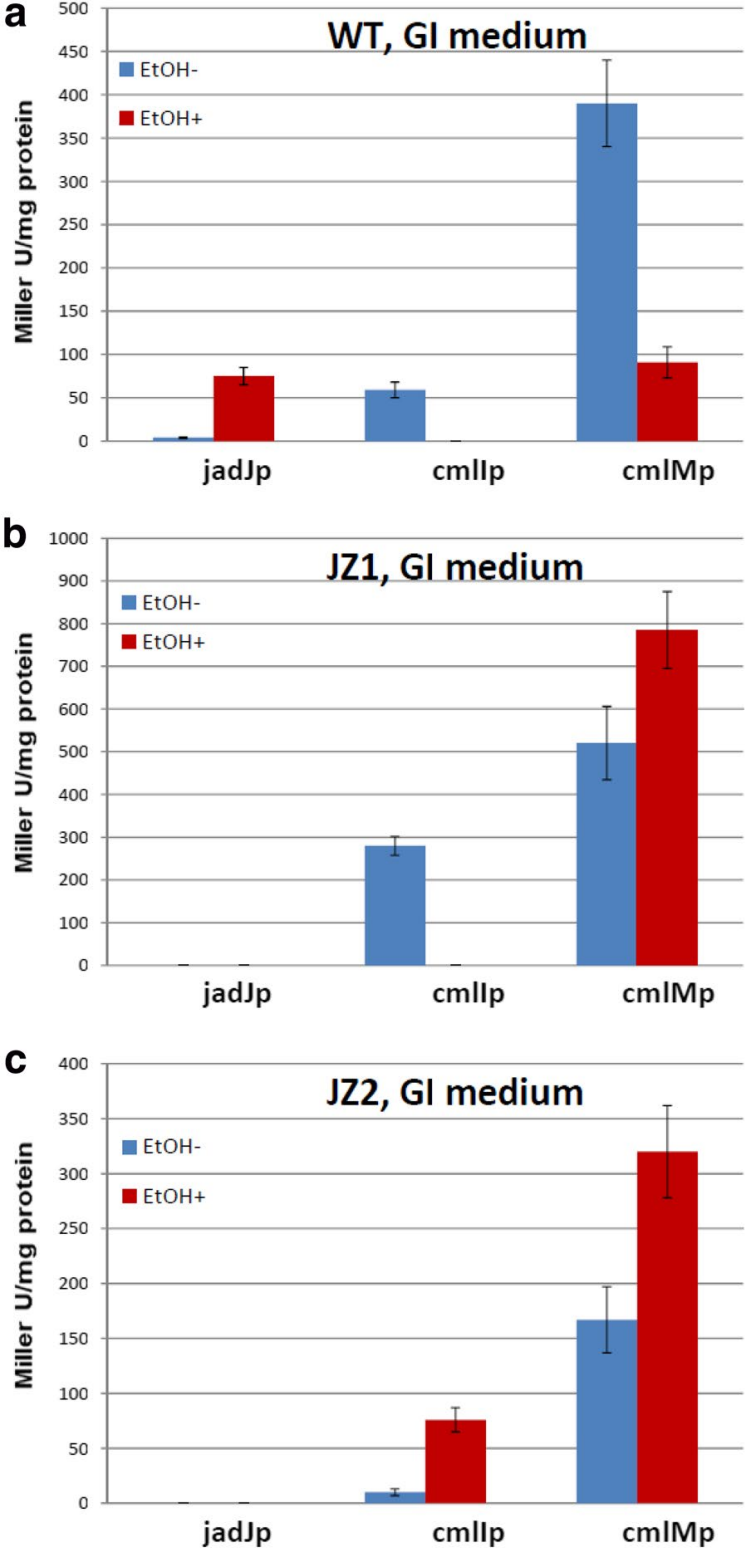
Effect of the ethanol shock on the activity of j*adJ*p, *cmlI*p and *cmlM*p promoters in the wild-type strain (**a**) and JZ2 (Δ*jadR1* Δ*cml*) mutant (**b**) grown in GI medium. Data presented are from 3 biological replicates, showing standard deviation

In order to correlate the earlier reports on down-regulation of Cml and up-regulation of Jad biosynthesis upon ES in GI medium with *cml* promoter activity, experiments with selected reporter strains were carried out. These experiments were also aimed at verification of the *S. venezuelae* behaviour that, in MYM medium, differed considerably from that reported previously for the GI medium. In the wild-type based strains grown in GI medium, in full accordance with the data from Xu et al. [5], ES strongly suppressed activity of the *cmlI*p and *cmlM*p promoters, while stimulating *jadJ*p (Fig. 5a). In the JZ1-based strains (devoid of JadR1) and without ES, *jadJ*p was silent while expression from both *cmlI*p and *cmlM*p was significantly stronger that in the wild type strain (Fig. 5b). ES completely abolished expression from the *cmlI*p promoter in this mutant, but had little effect on *cmlM*p. The situation was quite different in the JZ2 mutant devoid of both JadR1 and the *cml* gene cluster. For the JZ2-based strains, expression from the *cmlI*p and *cmlM*p promoters was clearly stimulated by the ES (Fig. 5c). The latter fact implied that the ability of the mutant to produce Cml plays a significant role in the regulation of *cml* cluster expression in response to ES. Interestingly, Cml yield after 48 h incubation in GI medium was 12–15 mg/L, which was considerably lower compared to the yield in MYM medium after ES.

## Discussion

Earlier reports from the Tan group revealed a complex regulatory network controlling biosynthesis of Jad and Cml in *S. venezuelae* (Fig. 1). According to these studies, performed using GI medium that normally supports Cml but not Jad biosynthesis, ES de-repressed transcription of *jadR1* encoding a DNA-binding protein that positively regulates Jad production while suppressing Cml biosynthesis. In the study by Xu et al. [5] JadR1 was shown to bind to the DNA region upstream of *cmlJ*, protecting ca 150 nt at a distance of 186 nt upstream of the *cmlJ* transcription start site (tss) from the action of DNase. According to the new study by Fernandez-Martinez [9] and our own analysis, this tss is located 76 nt upstream of the newly annotated *cmlM* gene, strongly suggesting that a promoter upstream of the latter gene drives expression of both *cmlM* and *cmlJ*, and possibly *cmlKS*. We found that the protection from DNase by JadR1 binding would extend into the *cmlI* gene, covering 120 nt of its 5′ end. Also, taking into account the overlap between start and stop codons of *cmlJ* and *cmlK*, respectively, and apparent gap between these genes in Fig. 6A in Xu et al. [5], it seems plausible that incorrect annotation of the genes’ boundaries in the *cml* cluster was responsible for this inconsistency. In the latter work, significantly higher production of Cml was observed in the *jadR1* deletion mutant grown in GI medium compared to the wild-type strain. This, together with the fact that JadR1 binds to the *cmlI*-*cmlM* intergenic region was the main evidence behind the conclusion about JadR1 being a repressor for the *cml* gene cluster.

Our results obtained in rich MYM medium that supports good growth of *S. venezuleae* contradict this conclusion, and suggest that mechanism of regulation of the *cml* gene cluster expression is more complex than previously assumed. ES strongly stimulated Cml biosynthesis both in the wild-type and *jadR1* deletion mutant JZ1, while production in the latter was significantly lower. These results correlated well with the data obtained in promoter fusion experiments, where ES stimulated expression from 3 *cml* promoters in the wild-type, while having a differential effect on these promoters in the JZ1 mutant (Fig. 4). Notably, no expression of a reporter gene from the *cmlR*p promoter could be detected in MYM medium both with and without ES in either wild-type strain or JZ1 mutant. Taken together, these data imply that in MYM medium, CmlR is not involved in controlling Cml biosynthesis, while JadR1 acts as a positive regulator only if culture is subjected to ES. At the same time, relatively low-level production of Cml upon ES in the JZ1 mutant points to the existence of additional regulatory protein(s) positively regulating Cml biosynthesis in response to ES.

Deletion of the *cml* gene cluster on *jadR1*-deficient background leading to Cml non-producing phenotype (mutant JZ2) specifically affected response of *cmlF*p promoter to ES, while having no effect on *cmlI*p and *cmlM*p. In JZ2 mutant, promoter for the *cmlF* gene encoding an efflux pump supposedly involved in resistance to Cml was stimulated by the ES, while it was repressed in the JZ1 mutant. The latter suggests that control over Cml biosynthesis may involve a feedback mechanism that involves binding of Cml to one or more regulatory proteins as a ligand, modulating their activity. It seems likely that such regulation involves JadR2 GBLR, a repressor of *jadR1*. Xu et al. has shown that Cml binding to JadR2 reduces its affinity to the jadR1p promoter, although its effect was not as strong as that of the Jad congener B [4].

Since our results contradicted earlier data obtained in GI medium, new experiments were conducted in this medium with selected promoters. Obviously, in GI medium and wild-type background the promoters reacted to ES as would have been expected from the published data: *jadJ*p promoter was stimulated by ES, while *cml* promoters were repressed (Fig. 5a). Moreover, higher transcriptional activity of *cml*Ip and *cmlM*p promoters on this medium in the *jadR1* deletion mutant support role of JadR1 as a repressor for *cml* cluster suggested by the Tan group. These data imply that regulation of Cml biosynthesis is highly medium-dependent, and JadR1 can act both as a repressor and as an activator for *cml* cluster. Moreover, promoters’ response to ES in GI medium was drastically different both in JZ1 (Δ*jadR1*) and JZ2 (Δ*jadR1*Δ*cml*) mutants. As expected from the JadR1′s role as a positive regulator of Jad biosynthesis, no expression was observed from the *jadJ*p promoter in these mutants with or without ES. In both JZ1 and JZ2 mutants ES had stimulating effect on the activity of *cmlM*p promoter (Fig. 5b, c). In contrast, *cmlI*p promoter was repressed in JZ1 mutant and stimulated in JZ2 mutant by ES, clearly pointing at the involvement of either Cml or its precursors in the regulation of *cml* gene expression.

Taking into consideration both new and available data, we suggest that the role of JadR1 in regulating Cml and Jad biosynthesis may depend on growth conditions and production of certain antibiotic congeners or precursors. When *S. venezuelae* is grown in the GI medium, JadR1 acts mostly as a repressor, although Xu et al. noticed unexpected decline of *cmlJ* expression at 48–72 h in their *jadR1* deletion mutant [5]. Wang et al. have demonstrated that JadR1 binds Jad congeners, and such binding inhibits its DNA binding activity toward the *jadJ*p promoter [4]. Interestingly, different Jad congeners had variable effects on JadR1, suggesting an intricate mechanism of autoregulation. Cml had no effect on JadR1 DNA binding [4], and it is thus conceivable that ES-induced production of certain metabolites in MYM medium modulates JadR1 activity with respect to its affinity for the *cml* promoters. Since Jad is not produced in the MYM medium irrespective of ES, it seems unlikely that these metabolites are represented by Jad congeners. Taking into account the effect of *cml* cluster deletion on the activity and ES response of certain promoters, it is conceivable that precursors in Cml biosynthetic pathway may play a role of such ligands. However, it is also possible that ES stimulates production of some other metabolites, not related to Jad or Cml, that somehow regulate JadR1 activity.

Cml production induction ratio in MYM medium after ES was ca 75 fold in the wild type strain, which is a rather impressive for antibiotic biosynthesis. Moreover, Cml yield in these conditions was >threefold higher compared to the Cml production in GI medium. Keeping in mind possible utility of *S. venezuelae* as a host for heterologous expression of secondary metabolite biosynthesis gene clusters [16], our results provide a basis for construction of ethanol-inducible expression system based on *cml* promoters. Indeed, inactivation of both Jad and Cml biosynthesis in the JZ2 mutant eliminated potential competition for the precursor pool upon heterologous expression of exogenous biosynthetic pathways. Also, *cmlI*p promoter, unlike in the wild-type strain, could be ca 13 fold stimulated by ethanol shock in JZ2. Such system represents a convenient “chassis” that can be used for controlled expression of antibiotic biosynthesis gene clusters in *S. venezuelae*.

## Conclusions

Although the effect of environmental stress on secondary metabolite biosynthesis is a widely accepted phenomenon, our knowledge of the molecular mechanisms behind it remains limited. In this study, we have provided a new insight into ethanol shock- and medium-dependent regulation of promoters from two different antibiotic biosynthesis gene clusters in *S. venezuelae*. Moreover, our results strongly support involvement of secondary metabolites or their biosynthetic precursors in this regulation. These findings pave the way for complete deciphering and re-design of stress-induced regulatory network in streptomycetes, which can assist in construction of a robust and controllable “chassis” for heterologous production of valuable bioactive compounds.

## Methods

### Strains, plasmids and growth conditions

Description of bacterial strains and plasmids used or generated during this study is provided in Table 2. *Streptomyces venezuelae* strains were maintained on ISP4 agar medium (Difco) supplemented, wherever needed, with 50 μg/mL apramycin. Starting cultures of *S. venezuleae* were prepared by inoculating ca 5^.^10^6^ spores in 10 mL liquid TSB medium (Oxoid), supplemented, wherever needed, with 50 μg/mL apramycin, followed by 16 h incubation at 30 °C in a 150 mL Erlenmeier flasks with shaking (220 rpm/min). 2 mL of the start culture were transferred to 250 mL buffled Erlenmeier flasks with 20 mL MYM [2] supplemented with 2.1 g/L MOPS (Sigma) or GI media [17] and incubated in the same conditions as for the starting cultures for 8 h. Next, 6 % v/v absolute ethanol (ES+) of sterile distilled water (ES−) were added, and the cultures were incubated for further 2, 8, 17, and 24 h (for GusA assays) or 48 h (Cml assay).

**Table 2:** Bacterial strains and plasmids used in this work

Bacterial strains	Genotype/phenotype	Source/reference
*Escherichia coli* DH5α	General cloning host: (*luxS supE44 ΔlacU169 (ϕ80 lacZΔM15) hsdR17, recA1, endA1, gyrA96, thi*-*1, relA1)*	BRL
*Escherichia coli* ET125671 (pUZ8002)	Mediates conjugative DNA transfer from RP4 oriT with helper plasmid pUZ8002 (KanR, CmR) Methylation deficient (*dam* ^−^ *, dcm* ^−^ *, hsdM* ^−^)	[16]
*Streptomyces venezuelae* ATCC10712 (ISP5230)	Wild type, chloramphenicol and jadomycin B producer (in response to disparate conditions)	ATCC
*S. venezuelae* JZ1	In-frame deletion of *jadR1*	This work
*S. venezuelae* JZ2	*cml* gene cluster deletion in the JZ1 mutant	This work
*S. venezuelae* SOK808::*cmlFp*	Wild type strain harbouring pSOK808::*cmlFp*	This work
*S. venezuelae* SOK808::*cmlIp*	Wild type strain harbouring pSOK808::*cmlIp*	This work
*S. venezuelae* SOK808::*cmlMp*	Wild type strain harbouring pSOK808::*cmlMp*	This work
*S. venezuelae* SOK808::*cmlRp*	Wild type strain harbouring pSOK808::*cmlRp*	This work
*S. venezuelae* SOK808::*jadJp*	Wild type strain harbouring pSOK808::*jadJp*	This work
*S. venezuelae* SOK808	Wild type strain harbouring pSOK808	This work
*S. venezuelae* JZ1 SOK808::*cmlFp*	JZ1 strain harbouring pSOK808::*cmlFp*	This work
*S. venezuelae* JZ1 SOK808::*cmlIp*	JZ1 strain harbouring pSOK808::*cmlIp*	This work
*S. venezuelae* JZ1 SOK808::*cmlMp*	JZ1 strain harbouring pSOK808::*cmlMp*	This work
*S. venezuelae* JZ1 SOK808::*cmlRp*	JZ1 strain harbouring pSOK808::*cmlRp*	This work
*S. venezuelae* JZ1 SOK808::*jadJp*	JZ1 strain harbouring pSOK808::*jadJp*	This work
*S. venezuelae* JZ2 SOK808::*cmlFp*	JZ2 strain harbouring pSOK808::*cmlFp*	This work
*S. venezuelae* JZ2 SOK808::*cmlIp*	JZ2 strain harbouring pSOK808::*cmlIp*	This work
*S. venezuelae* JZ2 SOK808::*cmlMp*	JZ2 strain harbouring pSOK808::*cmlMp*	This work
*S. venezuelae* JZ2 SOK808::*cmlRp*	JZ2 strain harbouring pSOK808::*cmlRp*	This work
*S. venezuelae* JZ2 SOK808::*jadJp*	JZ2 strain harbouring pSOK808::*jadJp*	This work

Plasmid	Genotype	Source/reference
pSOK201	pSG5 minimal replicon, Am^R^, *RP4 oriT*, *ColEI* replication origin	[22]
pSOK804	*ColEI* replication origin, Am^R^, *RP4 oriT,* VWB *attP* and *int*	[21]
pUWLoriT	pIJ101 minimal replicon, Thio^R^, Amp^R^, *RP4 oriT*, *ColEI* replication origin, ermE*p	[20]
pSOK806	*ColEI* replication origin, Am^R^, *RP4 oriT,* VWB *attP* and *int*, ermE*p	This work
pSOK808	*ColEI* replication origin, Am^R^, *RP4 oriT, attP*, *int*, *ermE*p*::gusA*	This work
pSOKjadD	Suicide plasmid for deletion of the *jadR1* gene	This work
pSOKcmlD	Suicide plasmid for deletion of the *cml* gene cluster	This work
p808cmlFp	*ColEI* replication origin, Am^R^, *RP4 oriT, attP*, *int*, *cmlF*p::*gusA*	This work
p808cmlIp	*ColEI* replication origin, Am^R^, *RP4 oriT, attP*, *int*, *cmlI*p::*gusA*	This work
p808cmlMp	*ColEI* replication origin, Am^R^, *RP4 oriT, attP*, *int*, *cmlM*p::*gusA*	This work
p808cmlRp	*ColEI* replication origin, Am^R^, *RP4 oriT, attP*, *int*, *cmlR*p::*gusA*	This work
p808jadJp	*ColEI* replication origin, Am^R^, *RP4 oriT, attP*, *int*, *jadJ*p::*gusA*	This work

*Escherichia coli* strains were maintained and manipulated as described elsewhere. Conjugation of recombinant plasmids from *E. coli* ET12567 (pUZ8002) into *S. venezuelae* was performed as described previously [18], with minor modifications. In particular, heat shock time was reduced to 5 min, and incubation of the conjugation plates was performed at room temperature for 16 h before applying antibiotic selection.

### DNA manipulation

General DNA cloning, PCR amplification and analyses were performed as described in Sambrook et al. [19]. For seamless assembly, DNA fragments were amplified using MasterAmp™ Extra-Long DNA Polymerase Mix and buffers (Epicentre, USA) and joined by Gibson ligation [14]. Primers for PCR were designed using j5 online software [20]. Recombinant constructs were verified by DNA sequencing. Genomic DNA was isolated using Wizard^®^ Genomic DNA Purification Kit (Promega, USA).

### Construction of promoter-probe plasmids

DNA fragment encompassing ermE*p promoter was amplified from the plasmid pUWLoriT [21] and inserted into the PCR-amplified pSOK804 vector [22] by Gibson assembly, generating plasmid pSOK806. The latter plasmid was used to clone a synthetic *gusA* gene codon-optimized for *S. venezuelae* (GenScript, USA) under control of *ermE*p*, yielding plasmid pSOK808. DNA fragments encompassing *cml* and *jadJ* promoters were PCR-amplified and used to replace ermE*p promoter in pSOK808 via Gibson assembly. Sequences of oligonucleotides used in PCR reactions are provide in Additional file 1: Table S1.

### Construction of *S. venezuelae* deletion mutants

1.5 kb DNA fragments flanking the *jadR1* gene and also encompassing 30 nt of the 5′ and 3′ ends of this gene were amplified from the genomic DNA of *S. venezuelae* and joined using Gibson assembly with a PCR-amplified part of pSOK201 [12] devoid of *Streptomyces* replication function. The resulting plasmid pSOKjadD was introduced into *S. venezuelae* wild-type strain by conjugation, and correct integration via single crossover confirmed by PCR. Second crossover mutants were selected after three rounds of overnight sub-culturing in TSB liquid medium in non-selective conditions, followed by growth and sporulation on ISP4. Apramycin-sensitive clones were selected by replica plating, and correct second crossover mutants confirmed by PCR. The Δ*jadR1* mutant was designated JZ1.

1.5 and 1.7 kb DNA fragments flanking central part of the *cml* gene cluster (genes *cmlE*-*cmlS*) were PCR-amplified and joined using Gibson assembly with a PCR-amplified part of pSOK201 [23] devoid of *Streptomyces* replication function. The resulting pSOKcmlD plasmid was introduced into the JZ1 mutant by conjugation and second crossover mutants selected as described above. The identity of the mutants was confirmed by PCR, and confirmed Δ*jadR1*Δ*cml* mutant was designated JZ2.

### Assays for chloramphenicol production and GusA activity

Cml extraction from cultures of *S. venezuelae* was done as described previously [24], but using 0.5 mL of culture supernatant for extraction, and the extracts were analysed by UHPLC–MS/MS. Analyses were performed with an ACQUITY UPLC system coupled to a Xevo TQ-S triple quadrupole mass spectrometer (Waters, Milford, MA, USA) equipped with an ESI source operating in negative mode. UHPLC–MS/MS data were acquired and processed using MassLynx software (v4.1) and TargetLynx application manager. The chromatographic column was a Waters ACQUITY UPLC^®^ BEH C18 (50 mm × 2.1 mm L × I.D. 1.7 µm) and the column manager was set to 55 °C. Mobile phase consisted of (A) water and (B) methanol, and flow rate was set at 0.300 mL/min. Conditions were kept constant at 70 % A for half a minute, then a linear gradient was programmed from 70 % A to 0 % A in 1 min, followed by 0 % A kept for an additional 1 min period, before the gradient was brought back to 70 % A in 0.10 min. Finally, the column was equilibrated for 0.90 min before starting a new injection. All samples and standards were dissolved in an equal amount of water and methanol. The injection volume was 2 µL. MS and MS/MS analyses were performed under constant ESI conditions. The capillary and source offset voltages were set at 2.5 kV and 50 V, respectively. The source temperature was maintained at 150 °C, desolvation gas temperature to 500 °C and flow rate was set at 1000 L/h. The cone gas flow rate was fixed at 150 L/h and the nebulizer gas flow maintained at 7 bar. The collision gas flow was set to 0.15 mL/min of argon. Cone voltages (CV), collision energies (CE) and MS/MS transitions (precursor and product ions) of chloramphenicol and chloramphenicol–d5 were optimized using Intellistart by infusing 100 nM standard solutions of each compound in an equal mixture of methanol and water at a flow rate of 20 µL/min.

Chloramphenicol was quantified by means of one selected precursor ion-product ion transition (m/z 321.1–151.6, CV = 25 V and CE = 20 eV), and its identity confirmed by three additional transitions (m/z 321.1–152.15, CV = 25 V, CE = 16 eV; m/z 321.1–194.2, CV = 25 V, CE = 12 eV, m/z 321.1–257.12 and CV = 25 V, CE = 12 eV). Chloramphenicol–d5 was monitored using the transition m/z 326.2–157.1 (CV = 25 V and CE = 20 eV). A 25 ms dwell time was selected for each transition.

GusA activity was measured as described by Fernandez-Martinez et al. [23], and calculated using the following equation: Miller units/mg total protein = 1000 × (OD_420_ − 1.75 × OD_550_)/time of reaction × volume of culture assayed × protein concentration mg/mL.

## Additional file

10.1186/s12934-016-0484-9 Supplementary tables (S1–S2) containing information on oligonucleotide primers used for amplification of DNA fragments.

## Abbreviations

ES: ethanol shock
Cml: chloramphenicol
Jad: jadomycin
GBLR: γ-butyrolactone receptor
