# Supplementary material for: Activation of chloramphenicol biosynthesis in Streptomyces venezuelae ATCC 10712 by ethanol shock: insights from the promoter fusion studies
Source: Microb Cell Fact. 2016 May 20;15:85. doi: 10.1186/s12934-016-0484-9 (PMC4875748; doi:10.1186/s12934-016-0484-9)
Supplement: Supplementary file 1 — 10.1186/s12934-016-0484-9 Supplementary tables (S1–S2) containing information on oligonucleotide primers used for amplification of DNA fragments. [file 12934_2016_484_MOESM1_ESM.doc]

**Supplementary Tables**

Sekurova et al. “Activation of chloramphenicol biosynthesis in *Streptomyces venezuelae* ATCC 10712 by ethanol shock: insights from the promoter fusion studies”

**Table S1.** Oligonucleotide primers used for amplification of DNA fragments for Gibson assembly of gene replacement constructs pSOKjadD and pSOKcmlD (see Table 1 in the main text).

| **Name** | **DNA sequence** | **Target DNA** | **Construct** |
| --- | --- | --- | --- |
| SOK201jr1_F | CGACATCAAGGACTGCGTAATCATGTCATAGCTGTTTCC | pSOK201 | pSOKjadD |
| SOK201jr1_R | GAGTCCGTGGTACAGGTCGACGGATCTTTTCC | pSOK201 | pSOKjadD |
| JR1del1-F | GATCCGTCGACCTGTACCACGGACTCCTCGACC | gDNA | pSOKjadD |
| JR1del1-R | GGATGGTGATCACCACTTCTACGGACGTCAGGC | gDNA | pSOKjadD |
| JR1del2-F | GTCCGTAGAAGTGGTGATCACCATCCGAGGC | gDNA | pSOKjadD |
| JR1del2-R | CTATGACATGATTACGCAGTCCTTGATGTCGCAGATGG | gDNA | pSOKjadD |
| SOK201jr1_F | CGACATCAAGGACTGCGTAATCATGTCATAGCTGTTTCC | pSOK201 | pSOKcmlD |
| SOK201jr1_R | GAGTCCGTGGTACAGGTCGACGGATCTTTTCC | pSOK201 | pSOKcmlD |
| cmlD1-F | GATCCGTCGACCTGTACCACGGACTCCTCGACC | gDNA | pSOKcmlD |
| cmlD1-R | GGATGGTGATCACCACTTCTACGGACGTCAGGC | gDNA | pSOKcmlD |
| cmlD2-F | GTCCGTAGAAGTGGTGATCACCATCCGAGGC | gDNA | pSOKcmlD |
| cmlD2-R | CTATGACATGATTACGCAGTCCTTGATGTCGCAGATGG | gDNA | pSOKcmlD |

**Table S2.** Oligonucleotide primers used for amplification of DNA fragments for Gibson assembly of promoter fusion constructs (see Table 1 in the main text).

| **Name** | **DNA sequence** | **Target DNA** | **Construct** |
| --- | --- | --- | --- |
| SOK804-F | GGTTGGTAGGATCCTGCAGGTCGACTCTAGAGG | pSOK804 | pSOK806 |
| SOK804-R | CCTGCACGAAGTGCGAAGTTCACCGAAGAGCG | pSOK804 | pSOK806 |
| ermEp-F | TCGGTGAACTTCGCACTTCGTGCAGGCGGTACC | pUWLoriT | pSOK806 |
| ermEp-R | AGTCGACCTGCAGGATCCTACCAACCGGCAC | pUWLoriT | pSOK806 |
| SOK806-F | CGGAAAGCAGTGACTGCAGGTCGACTCTAGAGGATCCG | pSOK806 | pSOK808 |
| SOK806-R | GGTTGGTGACTGCGATCCTACCAACCGGCACGATTGTCC | pSOK806 | pSOK808 |
| gusAS-F | GGTTGGTAGGATCGCAGTCACCAACCGCATCGATCG | gusA-S | pSOK808 |
| gusAS-R | CCTCTAGAGTCGACCTGCAGTCACTGCTTTCCGCCCTGCTGG | gusA-S | pSOK808 |
| 808cR-F | ACCTTTCCTGTCATCGATGACCATCGATCGAAGGAGAGTTCACCATGCTGA | pSOK808 | p808cmlRp |
| 808cR-R | TGCGTTCGCGATCCACGAAGTGCGAAGTTCACCGAAGAGCG | pSOK808 | p808cmlRp |
| cmlRp-F | TTCGCACTTCGTGGATCGCGAACGCATGGTGCAGCC | gDNA | p808cmlRp |
| cmlRp-R | ACTCTCCTTCGATCGATGGTCATCGATGACAGGAAAGGTGGTACGGC | gDNA | p808cmlRp |
| 808cF-R | ATGTAGTTGGAGCCATCGATCGAAGGAGAGTTCACCATGCTGA | pSOK808 | p808cmlFp |
| 808cF-F | TGTTCATGACGACCACGAAGTGCGAAGTTCACCGAAGAGC | pSOK808 | p808cmlFp |
| cmlFp-R | CTTCGCACTTCGTGGTCGTCATGAACACTCCTTCTCCGCG | gDNA | p808cmlFp |
| cmlFp-R | CCTTCGATCGATGGCTCCAACTACATCGCAGAAAGGGG | gDNA | p808cmlFp |
| 808cI-F | CCACAGACTTCGAGACCATCGATCGAAGGAGAGTTCACCATGCTGA | pSOK808 | p808cmlIp |
| 808cI-R | CCTTAAGGTGACGGAGCACGAAGTGCGAAGTTCACCGAAGAGC | pSOK808 | p808cmlIp |
| cmlIp-F | CTTCGCACTTCGTGCTCCGTCACCTTAAGGCCTCCG | gDNA | p808cmlIp |
| cmlIp-R | CCTTCGATCGATGGTCTCGAAGTCTGTGGATATCGGTCGCG | gDNA | p808cmlIp |
| 808cM-F | TAAGGTGACGGAGCATCGATCGAAGGAGAGTTCACCATGCTGA | pSOK808 | p808cmlMp |
| 808cM-R | ACAGACTTCGAGACCACGAAGTGCGAAGTTCACCGAAGAGC | pSOK808 | p808cmlMp |
| cmlMp-F | GGTGAACTTCGCACTTCGTGGTCTCGAAGTCTGTGGATATCGGTCGCG | gDNA | p808cmlMp |
| cmlMp-R | CCTTCGATCGATGCTCCGTCACCTTAAGGCCTCCG | gDNA | p808cmlMp |
| 808jJ-F | GGAGAAGGCGAGTCCATCGATCGAAGGAGAGTTCACCATGCTGA | pSOK808 | p808jadJp |
| 808jJ-R | GCTCGGACAGCGTCCACGAAGTGCGAAGTTCACCGAAGAGC | pSOK808 | p808jadJp |
| jadJp-F | CTTCGCACTTCGTGGACGCTGTCCGAGCGGACACC | gDNA | p808kadJp |
| jadJp-R | CCTTCGATCGATGGACTCGCCTTCTCCGTACCCG | gDNA | p808jadJp |

NB: gDNA – genomic DNA of *S. venezuelae* ATCC 10712.
